# Acute and Chronic Indigestion1Annual Address on Surgery, before the Lehigh Valley Medical Association, Easton, Pa., July 12, 1906.

**Published:** 1906-11

**Authors:** John B. Deaver

**Affiliations:** Philadelphia


### SELECTION.

### Acute and Chronic Indigestion.[1]

BY JOHN B. DEAVER, M. D., Philadelphia.

[*Boston Medical aud Surgical Journal.*]

AMONG the many heritages which civilisation brings in its wake, there is one disease so peculiar to civilized communities that its total absence may almost be considered pathognomonic of a semi-savage state of society. This disease is dyspepsia, and more particularly *chronic* dyspepsia. While acute dyspepsia is probably occasionally seen in the native Filipino and in the Hottentot or the inhabitants of Somaliland, it is unquestionably rare, and chronic dyspepsia is practically unknown. It remained for civilisation, with its overeating and underchewing tendencies, to fully develop these maladies, until at the present day they have assumed an importance in medicine and surgery second to no other affection.

1. Annual Address on Surgery, before the Lehigh Valley Medical Association, Easton, Pa., July 12, 1906.

It is, therefore, with no apologies for my subject that I have the honor of addressing you today. The only regret which can come to any speaker's mind, who has such a text for his remarks, is that the amount of time at his disposal, if not his own ability, must remain unequal to the task. Time is short and the art is long; and I must crave your indulgence if I seem to pass over in too cursory a manner some topics which may seem to deserve fuller consideration than it is possible for me to devote to them on this occasion.

There is no malady which is more easily diagnosed by the patient afflicted than is dyspepsia. It is an unheard of thing for an individual to have indigestion, either acute or chronic, and to remain unaware of the fact. Many a patient, as we all know, may suffer from extremely serious affections of the heart, of the kidneys, even of the liver and the lungs, without being at all aware of his precarious condition. But indigestion, produced by whatever cause, makes its presence felt, and usually it is a very unpleasant presence. And yet, what is remarkable about this self-assertive power of dyspepsia is, that its causes are so various, and at times so obscure. The variety of the diseases with which any other region of the body is afflicted is small in comparison with the affections of the abdominal organs. Inflammations affect all regions and every organ of the body; but with the exception of bronchitis and inflammations of the upper respiratory passages, inflammations of the digestive apparatus probably exceed in number the inflammations of every other portion of the body. Malignant growths, when occurring in the internal organs, affect the gastro-intestinal tract by preference,—with the possible exception of cancer of the uterus, which may almost be considered an external form of malignant disease. And so it is that dyspepsia, of one form or another, assumes the most important place in our nosological tables, because of its excessive prevalence. The subjoined table shows the relative frequency of the affections of the gastro-intestinal, the respiratory, the genito-urinary and the cardiovascular systems in the patients applying to our dispensaries.

Among 31,157 patients there were 4,924 or 15.8% in whom the gastro-intestinal tract was affected. In 7,193, or 23%, the respiratory system was diseased. In 819, or 2.7%, the genito-urinary system was diseased. In 903, or 2.8%, the cardio-vascular system was diseased.

And among children the proportion of gastro-intestinal cases is more than twice as great as diseases of the other systems, bearing about the same relation to the whole as in the case of adults.

Among 4,678 children there were 1,716, or 36.6%, in whom the gastro-intestinal tract was affected. In 1,392, or 29.7%, the respiratory system was diseased. In 119, or 2.5%, the genito-urinary system was diseased. In 47, or 1%, the cardiovascular system was diseased.

Now, of course the surgeon does not desire to monopolise the whole of that category of diseases described by the term dyspepsia. Selfish as he is thought to be and eager for more work as he has always shown himself, he is yet ready to recognise that there is a certain number of these cases,—indeed, a fairly large proportion of the whole,—which are in reality medical, in that no form of surgical treatment has yet been devised for their cure. Most cases of acute dyspepsia are undeniably medical; most cases of chronic dyspepsia can now be relieved by surgical means. And it is the duty of the progressive medical man, be he physician or surgeon, to make himself familiar with all such affections, and to be able to correctly advise his patients as to whether medical or surgical treatment is to be preferred in their particular case.

It is apparent, then, that diagnosis is in these diseases, as in all departments of medical science, the essential prerequisite to successful treatment, for although we must frankly acknowledge that there still exist some diseases which cannot be successfully treated even when their nature is known, it is even more certain that futile in the extreme are the misdirected efforts of that practitioner who attempts to relieve a patient, of the nature of whose malady he is totally ignorant.

In determining the cause of dyspepsia, two principles appear to me to be of importance: in acute affections trust to physical examination rather than the history, and in chronic diseases put more reliance on an accurately detailed clinical history than on the physical signs. I say, only, to put more confidence in the one than in the other; not to blindly trust one to the absolute exclusion of the other. Such a course could but be productive of disaster. But, as a general rule, physical signs are reliable in acute abdominal disorders, and in chronic diseases the clinical course of the disease frequently reveals the diagnosis unaided by other means. After these two cardinal methods of diagnosticating a malady have been used intelligently and exhaustively, then, and then only, will the attendant have recourse to mechanical means, such as blood examinations, skiagraphs and other laboratory methods. Most important is it, then, that physicians strive to recognise organic conditions in the stomach, and differentiate them from non-organic or functional disorders. It must be acknowledged that it is not only a waste of time and the resources of the patient to try to cure mechanical conditions medicinally or dietetically. Indigestion, acute or chronic, included under the name of dyspepsia, is a disturbance simply of the natural process of digestion. So soon as we have discovered a mechanical explanation for the condition, so soon can the cause be relieved by surgical intervention only. Persistent indigestion should call for investigation before we decide it is due to a functional disorder of the stomach. It is not to be understood that I would have you think that all serious disturbances of gastric digestion are due to anatomical changes in the stomach. Many cases of dyspepsia which I see are not due to organic disease of the stomach but to abnormal conditions of the circulation and nervous system. When the patient complains of pain following the ingestion of food, vomiting, vomiting of blood, witb or without loss of appetite, is pale and weak, and has lost flesh, and experiences relief by rest in bed and a restricted diet, this must mean, in almost every instance, organic disease of the stomach. Symptoms, such as fulness in the abdomen after eating, flatulence, heartburn, poor appetite and constipation, are those chiefly mentioned by patients and considered by physicians as found in dyspepsia. If such a patient is carefully examined and no organic disease can be detected, then we have to describe the case as one of functional disorder of the stomach.

It is unfortunate that investigation by test meal and the use of the stomach pump has not been of more value to us. In my experience I have no hesitancy in stating that they have no value where a differential diagnosis between organic and functional disease is to be made. Careful physical examination of the abdomen and other regions of the body, with a reliable history of the patient, is of much more moment than chemical examinations. Lavage, as now practised by many internists and stomach specialists, is not only overdone, but misleading, not only to the patient alone, but the physician as well.

While every pain in the abdomen should arouse the suspicion of appendicitis being present, the family physician is well aware that this disease is by no means the only cause of abdominal pain. But this I will say, that many and many a patient with appendicitis develops an abscess or a spreading peritonitis from no other cause than that the family physician has committed the crime of omitting a physical examination of the abdomen. Many hundreds of such cases have I seen. On the third or fourth day, when the attendant finds that anodynes and the liquid diet have not relieved the pain in the abdomen nor quieted the vomiting, then, alas, he recognises, by a physical examination too long delayed, that the pain which he had thought so trivial as perhaps even to have prescribed for it without seeing the patient,—a culpable treatment *in absentia*,—he realises, I repeat, that this seemingly trivial pain was in reality that most treacherous and malevolent disease, known by the name of appendicitis. Such a physician is more culpable even than one who, though aware of the true nature of the malady, yet attempts its cure by medical means. The result to the patient is very likely the same in either case; but carelessness is more to be blamed than ignorance.

Appendicitis is indeed a disease which of all other acute affections, ushered in by acute indigestion, can be most safely diagnosed by a physical examination. No patient with acute abdominal pain should ever be dismissed without having been submitted to palpation of the abdomen. The history is also of great importance, for by the sequence of symptoms, which in appendicitis is nearly pathognomonic,—by the pain first, then the vomiting, and at last the localised tenderness,—a vast horde of other abdominal affections may be readily excluded. When vomiting precedes the pain suspicions are at once aroused, and in most instances, as has been pointed out by Murphy, it will be found that the disease is not appendicitis. In children, the physician must consider simple overeating, with resultant acute gastritis—in such cases the history of overeating or of the ingestion of indigestible food, along with the negative physical examination, will make evident the true nature of the case. The swallowing of corrosive liquids or poisons must be constantly kept in mind, not only in children, who may be ignorant of the nature, but in adults, who may have ingested poison by mistake, in the dark, or with suicidal intent. The history is in all such cases of paramount importance, and is usually so clear as to leave no room for doubt. The various forms of intestinal obstruction must always be considered in patients with acute abdominal pain and vomiting. Frequently the acute pain of strangulation precedes the vomiting by a number of hours; and where the clinical history is negative for previous attacks of peritonitis or for abdominal operations, a careful search of the hernial rings will frequently reveal the presence of a strangulated hernia. In all conditions in which the peritoneum is involved in inflammation the salvation of the patient lies in early operation. Acute intestinal obstruction of all varieties,—strangulation by bands, strangulated hernia, internal hernia, intussusception or volvulus,—all are most successfully treated by operation, and the earlier the operation the surer is the cure. Internal strangulations can only be relieved by operative means, and early operation in strangulated hernia usually admits of radical cure of the rupture.

In the treatment of intestinal obstruction I desire to enter a plea for the old operation—Nelation's operation—of enterotomy. In these days of advanced surgery where the desire,—and a proper enough desire it is,—is to cure our patients by one operation alone, we are at times tempted to do too much, and in our desire to shorten our patient's confinement to bed we sometimes abbreviate his sojourn in this world. In cases of strangulation, where excision of a portion of the gut is required, our hope naturally is to be able immediately to restore the lumen of the bowel by an intestinal anastomosis. Usually this can be done with safety; but there still exist patients who are operated on so late that the toxemia—the copremia—from intestinal obstruction is so far advanced as to make any extensive operation very hazardous. It is in such patients, as I have said, that our attempt to complete an ideal operation results in the death of the patient. Such cases as these are greatly benefited by direct drainage of the intestinal tract, and to these Nelation's operation is particularly applicable. Even when the temporary establishment of a fecal fistula is not advisable, there can, I think, be no question that the distended bowel should be thoroughly emptied by some means before completing the operation. Either by emptying the proximal gut through the abdominal wound, before completing an intestinal anastomosis after resection of gangrenous bowel, or by evacuating the intestinal contents by means of massage of the bowel into a rectal tube passed high into the colon, I have in several instances successfully cured patients in whom toxemia from intestinal strangulation was marked.

Another prolific source of acute dyspepsia is disease of the biliary passages—infectious disease in practically all cases. Attacks of biliary colic, which are in many instances due rather to acute cholecystitis than to the passage of gallstones, are distinguished from recurrent attacks of appendicitis chiefly by the absolute freedom from indigestion during the intervals enjoyed by patients with gall-bladder disease. Patients with chronic, with recurrent, or with relapsing appendicitis are rarely entirely free from digestive disturbance. But it appears to be in some ways characteristic of gallstone patients that during the interval between their acute attacks they enjoy a most excellent digestion. They will relish lobster mayonnaise and deviled crabs with other highly seasoned food, and will digest their meals well; and then suddenly, out of the clear sky, as it were, and with no apparent relation to their meals or their indiscretions in diet, will come an attack of gallstone colic, with its intense nausea, its agonising pain, and its marked prostrations of strength.

Perhaps, before leaving the suject of acute indigestion, it will be well to mention the affection variously known as hyperemesis lactantium, congenital stenosis of the pylorus, infantile pyloric hypertrophy and similar titles. Whatever be its true nature, the symptomatology of this disease is fairly constant, and successful issues under operative treatment are rapidly becoming more frequent. Babies who formerly died of marasmus when a few months old, after a wretched existence characterised by persistent cumulative vomiting and by constipation, are now (thanks to the initiative of surgeons, such as Cautley and Dent in England, Nicoll in Scotland, Meinhardt Schmidt, and Trantenroth on the Continent, and Willy Meyer, Scudder, Quimby and others in this country) given a fair chance to assimilate their food and develop into healthy adults.

When we come to a consideration of the causes of chronic indigestion, it is necessary first to urge the postulate that persistent symptoms in any part of the body cannot be due to mere functional disturbances in that part. We cannot deny that through the intervention of the nervous system certain functional disorders may be produced in the portion of the body remote from the site of the actual structural change. Such conditions as the vomiting, which is frequently characteristic of brain tumor or of the abdominal pain and crises felt in Pott's disease and in locomotor ataxia are familiar instances of this truth. But surgeons do maintain and they are glad to see so able a physician as Professor Baus of Leeds[2] coming to the same conclusion, that persistent indigestion in the stomach, for example, must be due either to actual organic change in the stomach itself, or else to nervous or circulatory disturbances produced by disease in some other region of the body; that such persistent symptoms cannot be due to any functional disturbance of the organ affected, and that if disease of other regions of the body which might have a local influence on the functions of the gastro-intestinal tract can be excluded, we may with absolute and unerring certainty conclude that the local disease is organic in nature.

If, then, a patient complains to us of persistent or recurrent indigestion, which is relieved not at all or only temporarily by medical measures, we are surely justified in offering them as a solution of their structural disorders, some form of mechanical remedy; in short, some form of operation.

Chronic appendicitis does not, perhaps, today occupy so prominent a position in surgical discussions as it did a few years since, mainly because, I suppose, it is no longer so new. Its place has since been taken by gall-bladder and gastric affections, but it nevertheless is a disease widely prevalent, and very real and imminent in the sufferings it causes. But it is a disease which to

2. Lancet, 1906, i, 1588.

a very large extent is ignored or disbelieved in by the average physician, mainly because, I regret to say, one of the waves of injudicious surgery which periodically swell through our ranks was responsible for the removal of many an appendix which was not really productive of discomfort; the result being that physicians who saw patients supposed to have chronic appendicitis made not one whit better but rather worse by the removal of the organ,—the result being, I repeat, that physicians not unnaturally became skeptical as to the existence of such a morbid entity as chronic appendicitis.

Now it appears to me that in deciding from mere symptoms whether or not a patient has a chronic inflammation of his appendix, the surgeon should be very cautious; indeed, for my own part, unless such a patient presents a clear history of previous acute attacks, I am loth to operate; for although I have called attention to the fact that in cases of chronic indigestion the clinical history of the patient is usually more reliable than is a physical examination, yet it is absolutely essential to make a correct diagnosis to have either the clinical history or the physical examination absolutely explicit and conclusive if we would rely on one to the exclusion of the other. For a satisfactory diagnosis a certain definite sum total of factors is essential; and if we cannot obtain a sufficient quota from each of the two factors,—the history and the examination,—then we must obtain our whole quota from one to the exclusion of the other. Especially true is this in the case of chronic appendicitis. If the clinical history is explicit as to previous attacks of appendicitis, the diagnosis may safely be made even though the physical examination be negative. Or if the palpating hand can unmistakably feel a thickened, tender appendix, then, even though the patient never has had a frank attack of acute appendicitis, but merely presents a history of vague abdominal discomfort, possibly more or less localised at times to the region of the appendix, then, under these circumstances of positive physical signs, the clinical history may be disregarded, and the patient assured that operation will relieve him of his distress.

The danger, and it is not as remote as was formerly thought, of chronic disease of the appendix eventually becoming malignant or tuberculous in character, should not be lost sight of. I have recently[3] drawn attention to the fact that routine examination of appendices removed at operation reveals primary malignancy in as many as 2% (?) of the cases; and Professor Broca[4] has spoken of the danger of incipient tuberculous disease of the cecum being overlooked in children with chronic appendicitis.

3. Tr. Amer. Surg. Assoc., 1906.

4. Lancet, 1906, i, 1584.

So, likewise, with gall-bladder disease. Persistence or recurrence of symptoms indicates the existence of structural changes; and when once biliary calculi have formed, their attempted removal by medical means is always futile. As Dr. Kelly, the pathologist of the German Hospital, Philadelphia, has pointed out in his recent Mütter Lecture (delivered before the College of Physicians, Philadelphia, 1906), "We now know the thousands of gallstones said to have been passed by the bowel after the administration of olive oil and similar preparations are merely masses resembling gallstones in outward appearance, and due to the basely deceptive powers of the olive oil acquired in its passage through the intestinal tract." For not only are the stones commonly multiple, but many of them are so large that their passage through the ducts is a physical impossibility; and although in long standing cases of cholelithiasis large calculi are occasionally discharged into the bowel by way of cholecystoenteric fistulas, any physician who should anticipate with pleasure such a solution of the difficulty would be more fit for the days of medieval alchemy than for modern medicine or surgery. Moreover, even were all the calculi to be passed through the intestine by medical means, the infection of the gall-bladder would in most cases not only persist, but soon cause the formation of other calculi. For until physicians can combat as successfully as does surgery the infection which is the underlying condition in all cases of cholelithiasis, the less they have to do with acute complications of gallstone disease the better. As Dr. Kelly points out in his Mütter Lecture, "The favorable results of medicinal treatment in the vast majority of instances by no means indicate the cure of the disease, but merely the restoration of latency,"—and although this is "by no means an undesirable achievement," it is far from the *summum bonum* which is attained by operative means.

It may be stated, in passing, that in my own experience, as I believe in that of most surgeons, lesions of the pancreas are almost without exception due to pre-existent disease of the bile passages. Carcinoma of the pancreas may be an exception to this rule, but it is frequently so far advanced in its course before recognised that it is often impossible to determine where it had its primary origin, —whether in the bile duct or the pancreas itself; and its detection is in the end quite as often due to implication of the biliary apparatus as to manifest disturbances of the functions of the pancreas itself.

Chronic gastric dyspepsia has of late years afforded surgery a field for most signal triumph. I have on previous occasions quoted, and I take this opportunity of again quoting, Professor Kocher's statement in regard to the cure of gastric dyspepsia by surgical means, both because of their intrinsic value and because his name gives them added authority. Says Kocher,[5] "The majority of practitioners do not sufficiently realise what brilliant results are to be obtained by operative means in chronic affections of the stomach, commonly known as gastric catarrh. Not only can the numerous dangers of ulcerating affections of the stomach, such as hemorrhage, perforation, transition in cancer, be prevented, but the disease and its results may be so rapidly and certainly cured that the medical treatment of obstinate cases must be put in the background. . . The pain in the stomach disappears immediately after the operation. This is the invariable rule. . . The patient does not require to pay any further attention to the nature of his food. The vomiting disappears. . . . The bowels become regular. . . Repeated investigation of the gastric contents shows that there is a progressive improvement in the process of digestion; hyperacidity diminishes; if too little acid is present it becomes increased, a statement which is in agreement with Steudel, Carle and Fantino, Kautsch, Hartmann, Sipault and Mintz."

Even if we do not consider the relief of the patient's distressing symptoms,—pain, anorexia, flatulence, vomiting, and so forth, —as sufficient justification for operation, which it is, there yet remains the actual saving of life which operation procures by prevention of the later complications to which reference has been made. When we consider that under medical treatment 5% of patients with gastric ulcer die from hemorrhage, and 15% from perforations, the actual life saving value of operative treatment is evident from the fact that in skilled hands over 95% of patients recover from the operation, and that practically every one who recovers is permanently cured and relieved of all anxiety as to the occurrence of hemorrhage or perforation. And perforation and hemorrhage are not the only dangers to be feared in non-operated cases. It is becoming more and more widely recognised that in pre-existing ulcer, gastric cancer has its most frequent origin. Mumford and Stone[6] found this course to have been pursued by the ulcer in 41 out of 50 cases of gastric carcinoma recently studied by them; and Jedlicka[7] quotes Ssapesko as stating that among 100 cases of gastric carcinoma he found only 10 in which the cancer was not engrafted on a preceding ulcer. Jedlicka himself found that of the patients whose stomachs he resected for supposedly benign disease, more than one fourth gave microscopical evidence of carcinomatous changes in the ulcerated area. In deciding which patients are and which are not subjects

5. Op. Surg., Lond., 1903, p. 199.
6. Surgical Aspects of Digestive Disorders, 1905.
7. Op. Behandl. d. chrom. Magengeschwurs, Prag., 1904, s. 24.

of organic disease, I think reliance is to be placed first on the clinical history of the patient, then on the physical examination, and lastly, upon the results of laboratory methods of research. As I have already stated, in many cases the clinical history alone makes the diagnosis certain. Pain in a fixed spot in the epigastrium, soon after eating, this pain being relieved only when the stomach is emptied by vomiting or by the completion of digestion; the periodical but by no means regular occurrence of hematemesis; the presence of such symptoms in young and anemic females, —are all factors which speak for themselves of the presence of open ulcer, which render further investigation nearly unnecessary.

The existence of dilatation of the stomach due to pyloric stenosis can likewise usually be made without resort to laboratory methods when the patient presents a history of long-standing gastric dyspepsia, possibly attended years before by one or two attacks of hematemesis, but more recently characterised by persistent and cumulative vomiting,—by which I mean vomiting of food that has been accumulating in the stomach for days before, but which is only rejected when the dilated stomach has reached the limit of its capacity. In such patients an ordinary physical examination will often make evident the gastric dilatation; but if not, I prefer to dilate the stomach with air through a stomach tube, or by the administration of a Seidlitz powder in two portions, thus distending the stomach and rendering its outlines distinct to percussion. Such methods I think should only be employed in patients who are believed to have no acute ulceration present. In patients with open ulcer I consider the mere passage of a stomach tube dangerous, while distending the stomach with air might very probably cause perforation or provoke nearly fatal hemorrhage.

The detection of hyperchlorhydria I think in every way inferior to a determination of the motor power of the stomach. The ingestion of salol, with its subsequent detection in the urine, I do not regard as being so reliable a method for testing the motor power of the stomach as the use of a test meal, the remains of which are removed by lavage at the end of six hours. After the lapse of this period the normal stomach should be empty; but it should not be forgotten that increased motor power is not unfrequently present in patients with open ulcer and unobstructed pylorus, since the abnormal amount of hydrochloric acid usually present in these cases accomplishes gastric digestion of proteids in a precociously short time.

In the treatment of malignant disease in the stomach, the most important thing is to *cure in the precancerous stage.* I will not go so far as to state that gastric cancer, when so far developed as to be positively diagnosticated before operation, is necessarily eventually fatal, even though the patient's life may be prolonged, by resection, for a period of from one to eight years; but I will say, that with the knowledge we at present possess, no patient who presents symptoms of chronic gastric dyspepsia should be allowed to go on his way without prompt operation as a preventive of malignant disease. Because there is no manner of doubt that the absolute cure of a fully developed gastric cancer is one of the rarest things in surgery; it is, in fact, almost unknown. One or two patients have been known to survive gastrectomy for carcinoma for as long as eight years, and to be still in good health when last reported; but the patients who have most chance of ultimately remaining free from recurrence are those who have been subjected to gastrectomy before the diagnosis of malignancy could be made through the unopened abdominal wall.

I do not intend to argue here the advisability of operating on cases of gastric cancer on the ground that life will at any rate be prolonged and death rendered less unpleasant by this form of treatment, even though radical cure be not anticipated. I consider this a very sound reason for doing the operation; indeed, so far as statistics up to the present are concerned it is a reason for operating on these patients which has more in its support than any other; and in those rather unusual cases of gastric carcinoma in which the symptoms develop suddenly with no previous history of gastric dyspepsia, a patient past forty years of age becoming suddenly afflicted with anorexia, rapid emaciation and tumor formation,—in such cases, I repeat, it is the only reason which justifies operative interference at all, since in such patients permanent and total cure is all but unknown.

